# Sleep Arousal-Related Ventricular Repolarization Lability Is Associated With Cardiovascular Mortality in Older Community-Dwelling Men

**DOI:** 10.1016/j.chest.2022.09.043

**Published:** 2022-10-13

**Authors:** Sobhan Salari Shahrbabaki, Dominik Linz, Susan Redline, Katie Stone, Kristine Ensrud, Mathias Baumert

**Affiliations:** a School of Electrical and Electronic Engineering, The University of Adelaide, Adelaide, Australia; b Department of Cardiology, Maastricht University Medical Centre and Cardiovascular Research Institute Maastricht, Maastricht, the Netherlands; c Department of Cardiology, Radboud University Medical Center and Radboud Institute for Health Sciences, Nijmegen, the Netherlands; d Centre for Heart Rhythm Disorders, University of Adelaide and Royal Adelaide Hospital, Adelaide, Australia; e Department of Biomedical Sciences, Faculty of Health and Medical Sciences, University of Copenhagen, Copenhagen, Denmark; f Brigham and Women’s Hospital and Harvard Medical School, Boston, MA; g California Pacific Medical Center Research Institute, San Francisco, CA; h Department of Medicine and Division of Epidemiology and Community Health, University of Minnesota, Minneapolis, MN; i Center for Care Delivery and Outcomes Research, Minneapolis VA Health Care System, Minneapolis, MN

**Keywords:** all-cause mortality, cardiovascular mortality, QT variability index, sleep apnea, sleep arousal, ventricular repolarization, AHI, apnea-hypopnea index, CAD, coronary artery disease, CV, cardiovascular, CVD, cardiovascular disease, MI, myocardial infarction, PSG, polysomnogram, QTVi, QT variability index, REM, rapid eye movement, SD_QT_, SD of QT intervals, SD_RR_, SD of RR intervals

## Abstract

**Background:**

Sleep is fragmented by brief arousals, and excessive arousal burden has been linked to increased cardiovascular (CV) risk, but mechanisms are poorly understood.

**Research Question:**

Do arousals trigger cardiac ventricular repolarization lability that may predispose people to long-term cardiovascular mortality?

**Study Design and Methods:**

This study analyzed 407,541 arousals in the overnight polysomnograms of 2,558 older men in the Osteoporotic Fractures in Men sleep study. QT and RR intervals were measured beat-to-beat starting 15 s prior to arousal onset until 15 s past onset. Ventricular repolarization lability was quantified by using the QT variability index (QTVi).

**Results:**

During 10.1 ± 2.5 years of follow-up, 1,000 men died of any cause, including 348 CV deaths. During arousals, QT and RR variability increased on average by 5 and 55 ms, respectively, resulting in a paradoxical transient decrease in QTVi from 0.07 ± 1.68 to –1.00 ± 1.68. Multivariable Cox proportional hazards analysis adjusted for age, BMI, cardiovascular and respiratory risk factors, sleep-disordered breathing and arousal, diabetes, and Parkinson disease indicated that excessive QTVi during arousal was independently associated with all-cause and CV mortality (all-cause hazard ratio, 1.20 [95% CI, 1.04-1.38; *P* = .012]; CV hazard ratio, 1.29 [95% CI, 1.01 -1.65; *P* = .043]).

**Interpretation:**

Arousals affect ventricular repolarization. A disproportionate increase in QT variability during arousal is associated with an increased all-cause and CV mortality and may reflect ventricular repolarization maladaptation to the arousal stimulus. Whether arousal-related QTVi can be used for more tailored risk stratification warrants further study, including evaluating whether arousal suppression attenuates ventricular repolarization lability and reduces subsequent mortality.

**Clinical Trial Registration:**

ClinicalTrials.gov; No.: NCT00070681; URL: www.clinicaltrials.gov

**Take-home Points**

**StudyQuestion:** Is ventricular repolarization lability during sleep arousal associated with mortality?

**Results:** Cox proportional hazards models adjusted for confounders show that excessive QT variability during arousal is associated with an increased all-cause and CV mortality in older men.

**Interpretation:** QT variability may be an effective marker of cardiac maladaptation to arousal stimuli during sleep.

Sleep is fragmented by brief intrusions of unconscious wakefulness, so-called cortical arousals, which last between 3 and 15 s and are a normal feature of sleep.^1^ Sleep arousals can occur spontaneously or in response to sleep-disordered breathing, periodic limb movements, trauma, pain, temperature, light, and noise. Importantly, excessive nocturnal arousal burden (ie, > 8.5% and > 6.5% arousal time relative to total sleep time in men and women, respectively) is associated with long-term cardiovascular (CV) and all-cause mortality.^2^

CV responses to sleep arousal may explain, at least in part, the association between sleep arousals and mortality observed in epidemiologic studies.^2^ Irrespective of the underlying trigger,^3^ individual sleep arousals may elicit an acute transient activation of the autonomic nervous system and hemodynamic changes, including surges in sympathetic nerve activity and a combined volume and pressure overload.^4^ All of these arousal-related responses have been individually linked to worse CV outcomes,^3^ but none of them is currently incorporated into the clinical assessment of episodic arousals, which exclusively considers cortical activation via EEG traces on overnight polysomnography.^5^

Autonomic and hemodynamic alterations critically determine ventricular repolarization reflected in the T wave on an ECG. The QT interval, a global marker of ventricular repolarization duration, can be tracked on the ECG channel of a standard polysomnogram (PSG) to quantify the transient CV response to individual sleep arousals. Beat-to-beat variability in the QT interval captures transient dynamics in ventricular repolarization, yielding a simple, noninvasive marker of repolarization lability predictive of sudden arrhythmia death.^6^ In particular, when normalized to heart rate variability, the so-called QT variability index (QTVi) has been established as a powerful predictor of CV mortality in several patient populations.^7^ The quantification of arousal-related CV responses has the potential for a more disease-oriented and pathophysiology-based assessment of sleep-related abnormalities.^5^ It may provide the foundation for a better-tailored sleep arousal-specific risk stratification. Because high-fidelity computer algorithms for fully automated precise QT interval measurement are available,^8^^,^^9^ it could be incorporated into a clinical PSG assessment.

We hypothesized that the intensity of cardiac repolarization lability elicited by arousals identifies patients at risk for increased mortality. The objective of the current study therefore was to determine the QT variability in response to arousal in a large sample population of older men and its association with long-term CV and all-cause mortality.

## Study Design and Methods

### Study Population

We studied participants of the Osteoporotic Fractures in Men (MrOS) observational cohort study that enrolled 5,995 community-dwelling men aged > 65 years to investigate the epidemiology of osteoporosis in older men and identify the risk factors for fracture and bone loss.^10^, ^11^, ^12^ Of the 5,995 MrOS participants, 2,860 men in the main cohort did not participate in the sleep study visit (1,995 were unwilling to participate, 421 did not undergo screening because recruitment goals were met, 270 died prior to the sleep study visit, 150 were ineligible because they were receiving therapy for sleep apnea [CPAP or oxygen], and 24 were terminated prior to the sleep study visit).^13^ Thus, 3,135 men participated in the MrOS sleep study and completed an examination that included a clinic visit and overnight in-home overnight PSG, including a single-channel ECG.^13^^,^^14^ Of these men, 2,892 (92.2%) had technically adequate PSG, while another 196 men were excluded because of insufficient ECG quality. A flowchart of participants included in the study is presented in e-Figure 1.

### Follow-up

MrOS sleep participants were followed up via postcards and/or telephone every 4 months to survey for recent hospitalizations or medical treatment for CV disease (CVD) or clinically relevant arrhythmia requiring medical attention; the study had a *>* 99% response rate. A board-certified cardiologist verified all relevant medical records and supporting documents for centralized adjudication using a prespecified protocol.^15^ The death certificate and hospital records from the time of death were collected for fatal events. If a fatal event did not occur at the hospital, a proxy interview with the next of kin and the participant’s most recent hospitalization documents in the prior 12 months were collected. Only events confirmed by the adjudicator were included for analysis.

### In-Home Overnight PSG and Sleep Scoring

Sleep recordings were performed by using an unattended, portable in-home PSG over 1 night at the participant’s residence using the Safiro Sleep Monitoring System (Compumedics) for the MrOS sleep study. Trained staff members visited the participants to attach the sensors and electrodes and conduct overnight PSG. The setup included two central EEGs, bilateral electrooculograms, bilateral chin electromyogram, a bipolar ECG, nasal-oral thermistor, nasal flow via a pressure transducer and nasal cannula, abdominal and respiratory inductance plethysmography, finger pulse oximetry, bilateral leg movements by piezoelectric sensors, and body position.^14^

Research sleep technicians at a central sleep reading center, blinded to all other data, scored arousals along with other typical sleep events such as limb movements, respiratory events, and body movement, following the standard criteria defined by the American Academy of Sleep Medicine^1^ and described previously in detail.^13^^,^^16^ Interscorer and intrascorer reliability for all key indexes exceeded 0.90.

### Ventricular Repolarization Measurement

In 2,558 men, a total of 407,541 arousal episodes were observed, on average 159 events (27 per hour) per participant. For every arousal, beat-to-beat RR and QT intervals were automatically extracted from the ECG channel of the PSG using our validated two-dimensional signal-warping algorithm that tracks QT changes with high precision (e-Fig 2).^17^ Although QT variability can be observed on any ECG lead, leads with tall T waves, such as lead II, recorded during PSG, are preferable due to a better signal-to-noise ratio.^18^

The 15 s prior to every arousal onset were extracted to capture the baseline characteristics in the ECG. Because the cardiac response elicited by brief sympathetic activation, such as caused by arousal, largely diminishes within 10 s, and most arousals do not exceed a few seconds, the 15 s following arousal onset were captured. To investigate the effect of arousal on repolarization in detail, we measured both QT and RR intervals beat-to-beat across three 10 s time windows: (1) baseline period, –15 to –5 s prior to arousal onset detected in the central EEG channels; (2) arousal onset, –5 s prior to arousal onset to 5 s following arousal onset; and (3) postarousal onset, 5 to 15 s following arousal onset.

To quantify the ventricular repolarization dynamics prior to, during, and following arousal onset, we computed the QT variability index (QTVi):

$$QTVi=21n\frac{\frac{SD_{QT}}{M_{QT}}}{\frac{SD_{RR}}{M_{RR}}}$$

where SD_QT_, M_QT_, SD_RR_ and M_RR_ here represent the mean and SD of QT and RR intervals for each arousal. Because both QT and RR variability are measured in units of milliseconds, QTVi is dimensionless. Owing to the logarithm in the definition of QTVi, values < 0 indicate greater RR variability relative to QT variability, whereas QTVi > 0 represents higher QT variability than RR variability.

### Other Measures

All participants were required to attend a clinical interview and complete an enrollment form containing a questionnaire on medical history in advance of overnight PSG recordings. The participants’ race/ethnicity, BMI, and history of physician diagnosis of diabetes, hypertension, coronary artery disease (CAD), myocardial infarction (MI), congestive heart failure, transient ischemic attack, asthma, COPD, Parkinson disease, atrial fibrillation, and stroke were surveyed. Furthermore, participants reported smoking habits and alcohol consumption and completed the Physical Activity Scale for the Elderly questionnaire. Arterial BP was measured during the clinical visit. From overnight PSG, we derived the mean respiratory rate,^14^ the time of sleep spent below 90% oxygen saturation,^19^ the apnea-hypopnea index (AHI), the arousal index, arousal burden,^2^ and the periodic limb movement index.

### Statistical Analysis

QTVi values were divided into quartiles for Kaplan-Meier curve survival analysis and log-rank testing. Anthropometric data, lifestyle metrics, and medical history were compared by using dichotomized variables, *t* test, and χ^2^ test. A repeated measures analysis of variance was performed to evaluate variables prior to, during, and following the onset of arousal. We used restricted cubic splines with knots at the 5th, 35th, 65th, and 95th percentiles to explore the potential nonlinear association of the continuous variables with the outcome. Association between exposure variables (continuous and quartiles) and mortality were established with Cox proportional hazards models. The hazard ratio (HR) proportionality was determined by using cumulative sums of martingale residuals. Cumulative incidence function and Fine-Gray subdistribution hazard models were applied to predict CV death in the presence of competing risks of non-CV death. In all statistical tests, a *P* value of .05 was considered statistically significant.

MATLAB (R2020a, MathWorks) and Python libraries such as Lifelines and SciPy (Python Software Foundation) were used for computing and statistical analysis.

## Results

### Participant Characteristics

At the baseline visit, MrOS cohort participants were on average 76.3 ± 5.5 years old and had a BMI of 27.2 ± 3.8 kg/m^2^; almost one-half of the men were overweight (Table 1). About 16% of men had CAD/MI, and almost 6% had heart failure. Overnight PSG revealed that AHI, arousal index and arousal burden values were 19.9 ± 12.8 h^–1^, 25.1 ± 12.5 h^–1^, and 6.6% ± 3.2%, respectively.

**Table 1:** Cohort Characteristics of Osteoporotic Fractures (MrOS) Based on Arousal-Related QTVi Quartiles

Variable	All Subjects (N = 2,558)	QTVi < 0.42 (Q1-Q3) (n = 1,918)	QTVi ≥ 0.42 (Q4) (n = 640)	*P* Value
Age, y	76.3 ± 5.5	76.2 ± 5.5	76.5 ± 5.6	.256
Race				
White	2,336 (91.3)	1,739 (90.7)	597 (93.3)	.051
Black	79 (3.1)	58 (3.0)	21 (3.3)	.846
Asian	79 (3.1)	71 (3.7)	8 (1.2)	**.003**
Other	64 (2.5)	50 (2.6)	14 (2.2)	.658
Body weight				
BMI, kg/m^2^	27.2 ± 3.8	27.1 ± 3.7	27.5 ± 4.1	**.019**
Overweight	1,276 (49.9)	957 (49.9)	319 (49.8)	.982
Obese	525 (20.5)	393 (20.5)	132 (20.6)	.987
Cardiac assessment				
Atrial fibrillation	258 (10.1)	160 (8.3)	98 (15.3)	**< .001**
SBP, mm Hg	126.5 ± 17	126.4 ± 17	126.7 ± 17	.730
DBP, mm Hg	67.5 ± 9.4	67.4 ± 9.4	67.7 ± 9.3	.499
Lifestyle				
Smoking				
Never	1,019 (40)	764 (39.8)	255 (39.8)	.966
Past	1,489 (58.2)	1,116 (58.2)	373 (58.3)	.996
Current	50 (1.9)	38 (2.0)	12 (1.9)	.997
Current alcohol consumers	1,687 (66)	1,193 (62.2)	493 (77.0)	**< .001**
PASE score	146.4 ± 71.2	148.5 ± 71.6	140.2 ± 69.8	**.011**
Medical history				
Stroke	87 (3.4)	63 (3.3)	24 (3.8)	.663
CAD/MI	418 (16.3)	281 (14.7)	137 (21.4)	**< .001**
CHF	151 (5.9)	96 (5.0)	55 (8.6)	**.001**
TIA	233 (9.1)	163 (8.5)	70 (10.9)	.075
Asthma	202 (7.9)	151 (7.9)	51 (8.0)	.994
COPD	136 (5.3)	100 (5.2)	36 (5.6)	.764
HTN	1271(49.7)	938 (48.9)	333 (52)	.186
Diabetes	328 (12.8)	237 (12.4)	91 (14.2)	.249
Parkinson disease	27 (1.1)	18 (0.9)	9 (1.4)	.436
Overnight polysomnography				
AHI, h^–1^	19.9 ± 12.8	19.9 ± 13	20.0 ± 12.4	.956
AI, h^–1^	25.1 ± 12.5	25.0 ± 12.4	25.4 ± 12.7	.456
AB, %	6.6 ± 3.2	6.6 ± 3.4	6.6 ± 3.2	.728
PLMI, h^–1^	10.6 ± 10	10.5 ± 9.6	10.9 ± 11.1	.356
MRR, min^–1^	14.8 ± 1.9	14.7 ± 1.8	14.9 ± 1.7	**.049**
T90, min	14.2 ± 32.6	13.6 ± 31.5	15.9 ± 35.8	.118

Data are presented as mean ± SD or No. (%). Boldface indicates a *P* value with statistical significance. AB = arousal burden; AHI = apnea/hypopnea index; AI = arousal index; CAD = coronary artery disease; CHF = congestive heart failure; DBP = diastolic BP; HTN = hypertension; MI = myocardial infarction; MrOS = Osteoporotic Fractures in Men; MRR = mean respiratory rate; PASE = Physical Activity Scale for Elderly; PLMI = periodic limb movement index; QTVi = QT variability index; SBP = systolic BP; T90 = time of sleep spent below 90% oxygen saturation; TIA = transient ischemic attack.

Participants in the highest QTVi quartile were more likely to have a slightly greater BMI, consume alcohol, be less physically active, have a faster mean respiratory rate, and have a history of atrial fibrillation, congestive heart failure, and MI/CAD. e-Table 1 presents the men’s characteristics dichotomized on the highest quartile of QT and RR variance (SD_QT_ and SD_RR_).

### Repolarization Variability During Arousal

**Figure 1:**
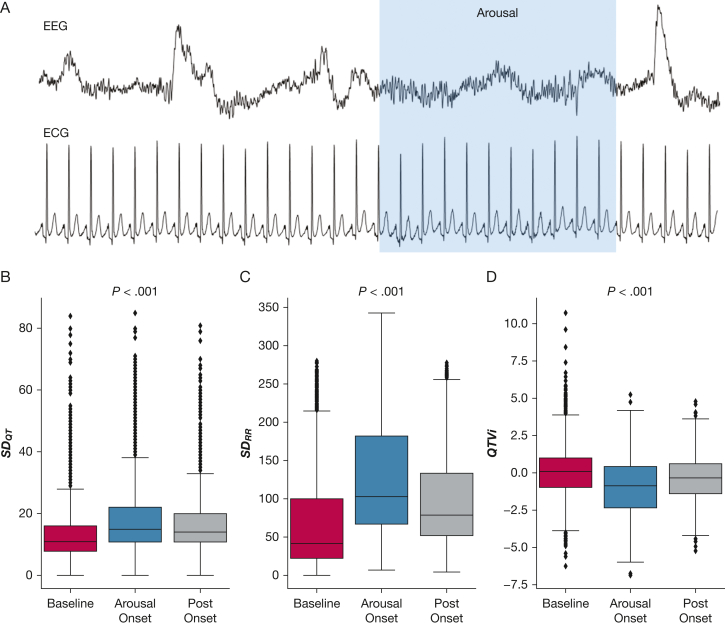
A, Graphical example of ECG and EEG activation during an arousal episode. Heart period and repolarization variability prior to (B), during (C), and following (D) the onset of arousal from sleep. P value indicates the repeated measures analysis of variance results. SD_QT_ and SD_RR_ = QT and RR variance; QTVi = QT variability index.

Figure 1A illustrates the transient ECG changes during cortical arousal. Across the entire cohort, QTVi significantly decreased from 0.07 ± 1.68 to –1.00 ± 1.68 at arousal onset and gradually recovered within 5 to 10 s following arousal onset (–0.39 ± 1.45*; P* < .001) (Fig 1D). Assessing the contributions of RR and QT interval variability individually, both significantly increased during arousal (baseline SD_QT_, 14 ± 10 ms; arousal SD_QT_, 19 ± 11 ms; baseline SD_RR_, 71 ± 66 ms; arousal SD_RR_, 126 ± 71 ms) (Figs 1B, 1C). The relative increase in RR variability was more pronounced than the QT variability (77% ± 7.5% vs 36% ± 10%), resulting in a transient paradoxical QTVi decrease.

Investigating the effect of arousal trigger on repolarization lability, respiratory event-related arousals caused significantly higher QTVi than nonrespiratory arousals (0.23 ± 0.89 vs –0.33 ± 0.68; *P* < .001); baseline QTVi was comparable (e-Fig 3).

Comparing arousal responses during rapid eye movement (REM) sleep with non-REM sleep, QTVi during arousal was higher in non-REM sleep than in REM sleep (–0.68 ± 0.59 vs –0.77 ± 0.61; *P* < .001); baseline QTVi was similar (e-Fig 4).

QTVi was only marginally influenced by arousal duration index (e-Fig 5) and arousal frequency (e-Fig 6).

### CV and All-Cause Mortality

End point data were available for 2,558 participants (e-Fig 1). During the mean ± SD follow-up period of 10.1 ± 2.5 years, 1,000 (39.1%) men died of any cause, including 348 (34.8%) confirmed CV deaths, 42 (4.7%) deaths from stroke, 249 (24.9%) deaths from cancer, and 81 (8.1%) pulmonary disease deaths. A total of 322 (32.2%) deaths were adjudicated as due to causes other than cancer, CVD, and pulmonary disease.

### Univariate and Multivariate Survival Analyses

**Figure 2:**
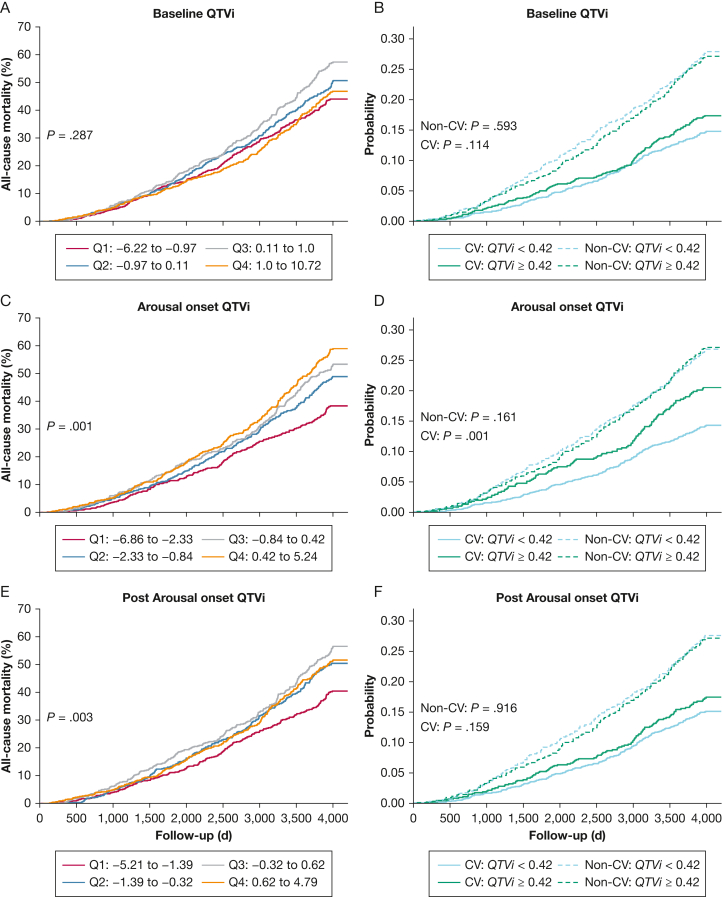
QTVi and mortality. All-cause mortality across QTVi quartiles at baseline (A), arousal onset (C), and postarousal onset (E). Cumulative incident function curves comparing the competing risk of CV and non-CV mortality of dichotomized QTVi at baseline (B), arousal onset (D), and postarousal onset (F). QTVi values were dichotomized using the fourth quartile of the arousal onset QTVi distribution. P value shows log-rank test results. CV = cardiovascular; Q1-Q4 = quartiles of the QT variability index for Baseline QTVi, Arousal onset QTVi and Post Arousal onset QTVi; QTVi = QT variability index.

Although prearousal QTVi was not significantly associated with mortality outcomes (Figs 2A, 2B), the QTVi response to arousal onset was modestly associated with increased overall mortality (Figs 2C, 2E). Subsequently, we dichotomized QTVi values on the fourth quartile of arousal onset QTVi to compare CV vs non-CV mortality risks. Augmented QTVi in response to arousal increased the probability of CV mortality by about 7% compared with men in the lower three QTVi quartiles (Fig 2D). The association between the highest QTVi quartile and CV mortality faded following arousal onset (Fig 2F). QTVi was not associated with non-CV mortality.

We developed univariate and multivariable Cox proportional regression models for QTVi prior to, during, and following arousal onset to assess associations with all-cause, CV, and non-CV mortality (Table 2). Continuous QTVi was significantly associated with CV mortality prior to, during, and following arousal onset in univariate Cox proportional hazards analysis. Associations were modest in magnitude (HR, 1.81-3.47 per 1 SD increase in QTVi). After adjusting the Cox proportional hazards regression model for age, BMI category, history of hypertension, MI/CAD, atrial fibrillation, congestive heart failure, stroke, diabetes, transient ischemic attack, Parkinson disease, asthma and COPD, drinking and smoking habits, arousal index, arousal burden, apnea/hypopnea index, mean respiratory rate, time spent with oxygen desaturation below 90%, and the average rate-corrected QT interval, continuous QTVi in response to arousal (but not QTVi at baseline) remained independently associated with all-cause mortality (HR, 1.47; 95% CI, 1.07-2.02; *P* = .029) and CV mortality (HR, 2.47; 95% CI, 1.43-4.25; *P* = .001), both expressed as per SD of QTVi. Forrest plot analysis (e-Fig 7) shows that the QT component rather than the RR component of QTVi is the primary driver of the association with CV mortality. Age (HR, 1.07; 95% CI, 1.05-1.10; *P* < .001), history of atrial fibrillation (HR, 1.79; 1.33-2.40; *P* < .001), CAD/MI (HR, 1.49; 95% CI, 1.14-1.90; *P* = .003), and diabetes (HR, 1.51; 95% CI, 1.12-2.00; *P* = .006), as well as mean respiratory rate (HR, 1.07; 95% CI, 1.00-1.10; *P* = .047), were significant CV outcome predictors in the model. We found no evidence of associations of QTVi prior to, during, or following arousal with the risk of non-CVD mortality.

**Table 2:** Association of QT and RR Time Intervals Variables Prior to, During, and Following Arousals With All-Cause and Cardiovascular Mortality

Variables	All-Cause Mortality (n = 1,000)				Cardiovascular Mortality (n = 348)				Noncardiovascular Mortality (n = 552)			
	Univariate Analysis		Multivariable Analysis		Univariate Analysis		Multivariable Analysis		Univariate Analysis		Multivariable Analysis	
	HR (95% CI)	*P* Value	HR (95% CI)	*P* Value	HR (95% CI)	*P* Value	HR (95% CI)	*P* Value	HR (95% CI)	*P* Value	HR (95% CI)	*P* Value
Prior to arousal												
QTVi (SD)	1.19 (0.86-1.64)	.287	1.20 (0.85-1.71)	.321	1.81 (1.06-2.18)	**.029**	1.66 (0.94-2.95)	.088	0.95 (0.63-1.41)	.789	1.01 (0.65-1.56)	.978
QTVi ≥ 0.42	1.03 (0.91-1.17)	.616	1.09 (0.95-1.25)	.238	1.19 (0.96-1.46)	.115	1.23 (0.98-1.55)	.074	0.96 (0.82-1.12)	.593	1.02 (0.86-1.21)	.861
Arousal onset												
QTVi (SD)	1.80 (1.35-2.41)	**< .001**	1.47 (1.07-2.02)	**.029**	3.47 (2.11-5.72)	**< .001**	2.47 (1.43-4.25)	**.001**	1.28 (0.89-1.82)	.169	1.12 (0.76-1.67)	.693
QTVi ≥ 0.42	1.25 (1.09-1.43)	**.001**	1.20 (1.04-1.38)	**.012**	1.49 (1.19-1.86)	**< .001**	1.29 (1.01-1.65)	**.043**	1.13 (0.95-1.34)	.161	1.11 (0.92-1.32)	.279
Postarousal onset												
QTVi (SD)	1.68 (1.15-2.44)	**.007**	1.37 (0.90-2.07)	.138	3.02 (1.59-5.72)	**< .001**	2.09 (1.04-4.18)	**.039**	1.23 (0.77-1.95)	.385	1.10 (0.66-1.85)	.763
QTVi ≥ 0.42	1.05 (0.92-1.20)	.455	1.02 (0.89-1.19)	.745	1.17 (0.94-1.46)	.159	1.05 (0.83-1.34)	.668	0.99 (0.84-1.17)	.916	1.01 (0.84-1.22)	.883

Multivariable analysis was adjusted for age, history of stroke, atrial fibrillation, myocardial infarction/coronary artery disease, congestive heart failure, transient ischemic attack, diabetes, hypertension, COPD, asthma and Parkinson disease, mean heart rate, mean respiratory rate, Physical Activity Scale for Elderly, systolic and diastolic BPs, time of sleep spent below 90% oxygen saturation, BMI, apnea-hypopnea index, arousal index, average corrected QT, arousal burden, and drink and smoking habit. QT variability index (QTVi) values were dichotomized at the 75th percentile of the arousal-onset QTVi. Boldface indicates a *P* value with statistical significance. HR = hazard ratio.

**Figure 3:**
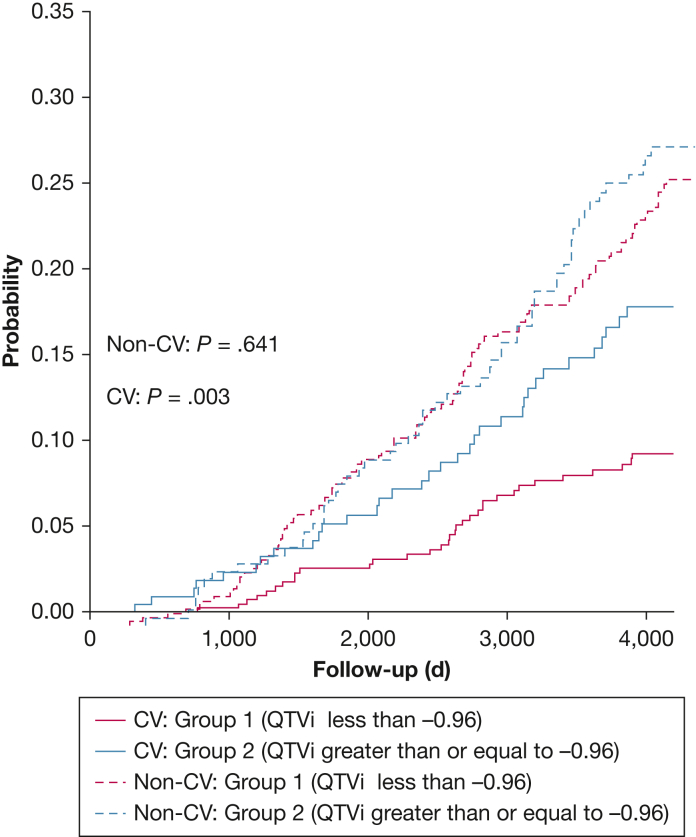
The exposure-response relationship of arousal-related QTVi and CV mortality adjusted for age, history of stroke, myocardial infarction/coronary artery disease, atrial fibrillation, congestive heart failure, diabetes, hypertension, COPD, asthma, mean heart rate, mean respiratory rate, Physical Activity Scale for Elderly, systolic and diastolic blood pressures, time of sleep spent below 90% oxygen saturation, BMI, apnea-hypopnea index, arousal index, average corrected QT, arousal burden, and drink and smoking habit. CV = cardiovascular; QTVi = QT variability index.

The exposure-response analysis shows the nonlinear association of QTVi at arousal with CV mortality adjusted for confounders (Fig 3). The risk of CV mortality gradually increased with an increase in QTVi beyond –1. e-Figure 8 further illustrates the association between QTVi quartiles and CV mortality. To remove the effect of atrial fibrillation from the analysis, we subsequently only considered participants with no history of atrial fibrillation. The Cox model confirmed the significant association between increased QTVi at arousal onset and CV mortality (continuous HR, 1.26 [95% CI, 1.12-1.43; *P* < .001]; dichotomous HR, 1.32 [95% CI, 1.02–1.69; *P* = .036) (e-Table 2). In particular, the QT component of QTVi, age, CAD/MI, diabetes, physical activity, and AHI were predictive in people with no history of atrial fibrillation (e-Fig 9).

### Subgroup Analyses

**Figure 4:**
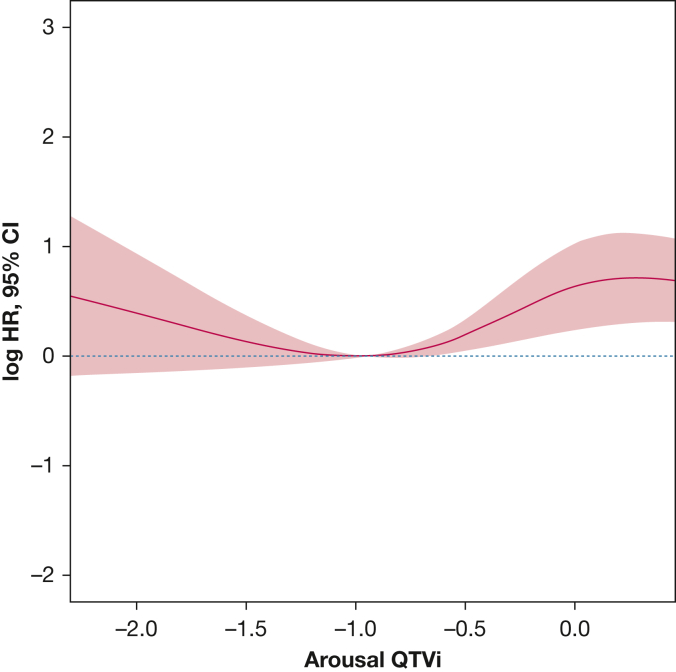
Cumulative incident function curves compare the competing risk of cardiovascular and noncardiovascular mortality in the men of the first quartile of baseline QTVi. Group 1 represents men whose QTVi values following arousal onset were still in Q1 (QTVi less than –0.96); Group 2 represents participants whose QTVi shifted to upper quartiles (Q2-Q4) following arousal onset. The P value indicates log-rank test results. HR = hazard ratio; QTVi = QT variability index.

To validate the effect of arousal on ventricular repolarization lability and its association with mortality, we investigated the subgroup of men whose QTVi was in the lowest quartile during baseline; that is, considered normal but increased beyond the first quartile during arousal. Men who developed larger QTVi responses during arousal were older; had a higher BMI, lower physical activity, and a history of atrial fibrillation; and were more likely to have severe sleep apnea (Table 3). The risk of CV death was about 9% greater in men whose QTVi increased to arousal (*P* = .007) (Fig 4). Univariate and multivariable analyses confirmed the association between an arousal-related QTVi increase and CV mortality (HR, 2.02 [95% CI, 1.25-3.26; *P* = .004]; HR, 1.89 [95% CI, 1.14-3.14; *P* = .013]) (Table 4). There was no significant association between adjusted QTVi and non-CV mortality in this subgroup.

**Table 3:** Characteristics of the Sample With a Baseline QTVi Within the First QTVi Quartile

Variable	All Subjects (N = 640)	Arousal QTVi Less Than –0.96 (Q1) (n = 417)	Arousal QTVi Equal to Greater Than –0.96 (Q2-Q4) (n = 233)	*P* Value
Age, y	76.2 ± 5.4	75.8 ± 5.2	76.8 ± 5.6	**.025**
White	579 (90.5)	372 (89.2)	207 (92.8)	.179
African-American	19 (3.0)	10 (2.4)	9 (4.0)	.358
Asian	26 (4.1)	22 (5.3)	4 (1.8)	.055
Other	16 (2.5)	13 (3.1)	3 (1.3)	.270
Body weight				
BMI, kg/m^2^	26.8 ± 3.6	26.5 ± 3.5	27.2 ± 3.6	**.011**
Overweight	315 (49.2)	213 (51.1)	102 (45.7)	.228
Obese	124 (19.4)	69 (16.5)	55 (24.7)	**.018**
Cardiac assessment				
Atrial fibrillation	44 (6.9)	13 (3.1)	31 (13.9)	**< .001**
SBP, mm Hg	127.0 ± 16.2	126.7 ± 15.9	127.5 ± 16.6	.561
DBP, mm Hg	67.3 ± 9.4	67.4 ± 9.1	67.1 ± 9.8	.642
Lifestyle				
Smoking				
Never	237 (37)	150 (36.0)	87 (39.0)	.501
Past	388 (60.6)	257 (61.6)	131 (58.7)	.531
Current	15 (2.3)	10 (2.4)	5 (2.2)	.881
Current alcohol consumers	431 (67.3)	281 (67.4)	150 (67.3)	.954
PASE score	152.5 ± 69.8	156.5 ± 70.6	144.9 ± 67.6	**.049**
Medical history				
Stroke	23 (3.6)	15 (3.6)	8 (3.6)	.829
CAD/MI	88 (13.8)	59 (14.1)	29 (13.0)	.779
CHF	23 (3.9)	13 (3.1)	12 (5.4)	.232
TIA	57 (8.9)	32 (7.7)	25 (11.2)	.177
Asthma	60 (9.4)	35 (8.4)	25 (11.2)	.306
COPD	33 (5.2)	16 (3.8)	17 (7.6)	.060
HTN	313 (48.9)	197 (47.2)	116 (52.0)	.285
Diabetes	87 (13.6)	55 (13.2)	32 (14.3)	.774
Parkinson disease	6 (0.9)	4 (1.0)	2 (0.9)	.724
Overnight polysomnography				
AHI, h^–1^	18.7 ± 12.3	18.0 ± 12	20.0 ± 12.7	**.049**
AI, h^–1^	24.0 ± 11.6	24.0 ± 11.1	24.0 ± 12.5	.997
AB, %	6.3 ± 3.1	6.2 ± 2.9	6.6 ± 3.5	.077
PLMI, h^–1^	10.8 ± 8.4	11.0 ± 8.8	10.4 ± 7.7	.414
MRR, min^–1^	14.7 ± 1.9	14.6 ± 1.8	14.7 ± 1.9	.501
T90, min	12.2 ± 31.5	11.0 ± 29.6	14.5 ± 34.8	.186

Data are presented as mean ± SD or No. (%). Boldface indicates a *P* value with statistical significance. AB = arousal burden; AHI = apnea/hypopnoea index; AI = arousal index; CAD = coronary artery disease; CHF = congestive heart failure; DBP = diastolic BP; HTN = hypertension; MI = myocardial infarction; PASE = Physical Activity Scale for Elderly; PLMI = periodic limb movement index; MRR = mean respiratory rate; QTVi = QT variability index; SBP = systolic BP; T90 = time of sleep spent below 90% oxygen saturation; TIA = transient ischemic attack.

**Table 4:** All-Cause and Cardiovascular Mortality Risk in Men of the First Quartile of Baseline QTVi

Variables	All-Cause Mortality				Cardiovascular Mortality				Noncardiovascular Mortality			
	Univariate Analysis		Multivariable Analysis		Univariate Analysis		Multivariable Analysis		Univariate Analysis		Multivariable Analysis	
	HR (95% CI)	*P* Value	HR (95% CI)	*P* Value	HR (95% CI)	*P* Value	HR (95% CI)	*P* Value	HR (95% CI)	*P* Value	HR (95% CI)	*P* Value
Continuous QTVi	1.05 (0.95-1.16)	.306	0.98 (0.89-1.08)	.743	1.18 (0.99-1.41)	.068	1.12 (0.93-1.35)	.217	1.00 (0.89-1.13)	.967	0.92 (0.82-1.04)	.194
QTVi greater than –0.96	1.31 (1.01-1.70)	**.047**	1.12 (0.86-1.48)	.391	2.02 (1.25-3.26)	**.004**	1.89 (1.14-3.14)	**.013**	1.08 (0.78-1.49)	.641	0.91 (0.65-1.27)	.579

Multivariable analysis was adjusted for age, history of stroke, atrial fibrillation, myocardial infarction/coronary artery disease, congestive heart failure, transient ischemic attack, diabetes, hypertension, COPD, asthma and Parkinson disease, mean heart rate, mean respiratory rate, physical activity scale for elderly, systolic and diastolic BPs, time of sleep spent below 90% oxygen saturation, BMI, apnea-hypopnea index, arousal index, average corrected QT, arousal burden, and drink and smoking habit. Boldface indicates a *P* value with statistical significance. QTVi = QT variability index.

## Discussion

To the best of our knowledge, the current study is the first to show that arousal-related transient changes in ventricular repolarization contribute to high repolarization lability reflected by changes in arousal-related QTVi. Irrespective of the cause of sleep arousal, arousal-related QTVi identified a group of older men at higher risk of long-term all-cause and CVD mortality.

Studies have shown changes in ventricular repolarization in patients with conditions typically associated with increased arousal burden. For example, patients with sleep-disordered breathing have an increased arousal burden, a prolonged QT interval, and increased QT interval variability,^20^ and they experience increased rates of premature ventricular contractions, all of which might increase their risk^21^^,^^22^ for sudden cardiac death.^3^ Herein, we extend this finding by showing a direct temporal relationship between episodic sleep arousals and transient dynamics in ventricular repolarization. In a detailed beat-to-beat analysis, we found that QT and RR interval variability rapidly increased at arousal onset and gradually decreased subsequently. Of note, the relative increase in RR variability was more pronounced than the QT variability increase, resulting in a transient paradoxical QTVi decrease. Irrespective of the cause of sleep arousal, higher arousal-related QTVi was independently associated with an increased risk of long-term mortality. This further supports the observation by Schmidt et al^23^ that QTVi increases in REM sleep predict death from CVD in the Sleep Heart Health Study. We assessed explicitly arousal-related QTVi, which focuses on responses in ventricular repolarization to arousals rather than non-specific QT dynamics during different sleep stages. Although there are no normative data on arousal-related QTVi yet available, and there is considerable variation in QTVi values reported in the literature in general,^24^ owing to differences in measurement, an increase in QTVi from –2.33 to 0.43 between the first and third quartiles (Fig 2) would seem significant. Effect sizes reported in the risk stratification literature are of similar magnitude.

Interestingly, QTVi prior to arousal onset (baseline) was not significantly associated with increased long-term mortality and was comparable between REM and non-REM sleep. The association between arousal-related QTVi and long-term mortality was independent of the baseline QTVi, suggesting the involvement of arousal-related mechanisms. We speculate that arousal-related autonomic and hemodynamic responses may detrimentally affect the heart and transiently expose the individual to increased CV risk during sleep.^3^ Interestingly, arousals induced by simulated obstructive respiratory events in healthy pigs transiently dissociate ventricular electromechanical coupling, creating a dynamic arrhythmogenic substrate during sleep.^25^ Our data show higher QTVi during respiratory arousals than other types of arousal, indicative of pronounced repolarization lability following respiratory events. The duration of the arousal only marginally affected QTVi.

Frequent arousal results in significant sleep fragmentation and circadian rhythm disturbance. In turn, they contribute to increased CV mortality by various mechanisms involving autonomic nervous system activation, nocturnal BP, and heart rate increases.^3^ Long-term exposure to repeated arousal-related pathophysiological conditions may create a detrimental substrate contributing to CV long-term mortality. QTVi may also represent a risk marker in some patients. Concomitant conditions associated with increased arousal burden, such as sleep-disordered breathing, shift work,^26^ or sleep deprivation^27^ due to nocturnal noise pollution, have all been shown to increase all-cause and CV mortality. Moreover, irregular sleep duration^28^ and timing increase CVD risk independent of traditional CVD risk factors and sleep quality and quantity.^3^^,^^29^^,^^30^ Indeed, participants in the highest QTVi quartile were more likely to report a history of CAD, MI, or congestive heart failure. Thus, the association between elevated QTVi and long-term CV may be caused by excessive sympathetic outflow to the ventricles as part of the normal arousal response^31^ or indicate structural heart disease or a combination of both. Because we adjusted the Cox regression model for self-reported CAD/MI and congestive heart failure, excessive sympathetic outflow following arousal is likely to contribute to elevated QTVi and possibly a key driver mediating the relationship with long-term CV mortality. Indeed, the subgroup analysis of men with strong QTVi arousal responses (Table 2) suggests an essential role of autonomic nervous system activation. The relationship between QT variability and sympathetic outflow has been documented using cardiac noradrenaline spill-over measurement,^32^ muscle sympathetic nerve activity, or pharmacologic adrenergic receptor activation.^24^ In particular, rhythmic repolarization changes are linked with sympathetic drive.^33^^,^^34^

Several consensuses^3^^,^^5^^,^^35^ and scientific statements propose a more disease-oriented and pathophysiology-based assessment of sleep-related abnormalities. Extending the continuous effort to improve the evaluation of sleep-disordered breathing severity by incorporating apnea-related hypoxemia^17^^,^^36^^,^^37^ and heart rate responses,^38^ we introduce arousal-related QTVi to quantify cardiac repolarization responses to arousal. QTVi is a robust, established ECG-derived parameter that yields a simple, noninvasive measure of repolarization dynamics predictive of sudden arrhythmia death^3^^,^^39^ and could be integrated into analysis software packages to analyze clinical PSGs. In addition, wearable devices^40^ that can provide surrogate parameters of arousal burden and ECG recordings during sleep could supply valuable data on periodic repolarization dynamics to apply this approach more widely.^41^^,^^42^ Whether a routine assessment of arousal-related cardiovascular responses such as arousal-related QTVi improves the clinical evaluation of arousals and results in a better and more tailored sleep arousal-specific risk stratification of a patient^5^ remains to be established. In addition, its utility in guiding personalized interventions to reduce arousal-related risks, such as managing the underlying conditions and lifestyle changes, warrants further study.

The current study had certain limitations. Participants were predominately white men and were predominantly older. Hence our findings cannot be extrapolated to women, other races, or younger individuals. Further studies, including men and women, can help delineate the relationship between cardiac arousal response, sex, and mortality. Our observations are based on a single night. Repeated studies over multiple nights will be required to shed light on day-to-day variations and reproducibility. Also, baseline exposure to various conditions was self-reported rather than systematically ascertained through medical records or direct measurement. We did not consider the possible confounding effects of medications and did not examine the cause of arousal. *P* values were not adjusted for multiple testing. In addition, in line with the American Academy of Sleep Medicine scoring rules, cortical but not subcortical arousals were considered. The current study investigated the association between arousal-related QTVi response and mortality. Individual arousal causes were not explicitly modeled, although we adjusted the regression models for arousal burden and common arousal triggers such as sleep-disordered breathing (eg, AHI, time of sleep spent below 90% oxygen saturation). Our observation of transient changes in ventricular repolarization around arousals does not prove a causal relationship.

## Interpretation

Sleep arousal-related variability in ventricular repolarization, quantified by QTVi, is associated with long-term all-cause mortality, primarily due to higher CV mortality, in older community-dwelling men. Further intervention studies targeting sleep arousal are warranted to investigate whether sleep arousal-related QTVi represents a modifiable risk marker of underlying disease or a modifiable risk factor.

## Funding/Support

This study was supported by a grant from the Australian Research Council [DP0663345]. The MrOS study and the Study of Osteoporotic Fractures (SOF) are supported by National Institutes of Health (NIH) funding. The following institutes provided support: The National Institute on Aging, the National Institute of Arthritis and Musculoskeletal and Skin Diseases, the National Center for Advancing Translational Sciences, and NIH Roadmap for Medical Research [U01 AG027810, U01 AG042124, U01 AG042139, U01 AG042140, U01 AG042143, U01 AG042145, U01 AG042168, U01 AR066160, and UL1 TR000128]. The National Heart, Lung, and Blood Institute provided funding for the MrOS sleep ancillary study [R01 HL071194, R01 HL070848, R01 HL070847, R01 HL070842, R01 HL070841, R01 HL070837, R01 HL070838, and R01 HL070839] and the National Sleep Research Resource [R24-HL-114473]. The SOF sleep study was supported by grants AG021918, AG026720, AG05394, AG05407, AG08415, AR35582, AR35583, AR35584, R01 AG005407, R01 AG027576-22, 2 R01 AG005394-22A1, 2 R01 AG027574-22A1, HL40489, and T32 AG000212-14. S. R. was supported in part by the NIH [R35HL135818].

## Financial/Nonfinancial Disclosures

None declared.

## Supplementary Data

e-Online Data
