# Supplementary material for: Sleep Arousal-Related Ventricular Repolarization Lability Is Associated With Cardiovascular Mortality in Older Community-Dwelling Men
Source: Chest. 2022 Oct 13;163(2):419–32. doi: 10.1016/j.chest.2022.09.043 (PMC9899642; doi:10.1016/j.chest.2022.09.043)
Supplement: e-Online Data [file mmc1.docx]

**Online Supplement**

Sleep arousal-related ventricular repolarisation lability is associated with all-cause and cardiovascular mortality in older community-dwelling men

Sobhan Salari Shahrbabaki, PhD^1*^; Dominik Linz, MD, PhD^2-5*^; Susan Redline, MD, MPH^6^; Katie Stone, PhD^7^; Kristine Ensrud, MD, MPH^8,9^; Mathias Baumert, PhD^1^.

^1^School of Electrical and Electronic Engineering, The University of Adelaide, Adelaide, Australia.

^2^Department of Cardiology, Maastricht University Medical Centre and Cardiovascular Research Institute Maastricht, Maastricht, the Netherlands.

^3^Department of Cardiology, Radboud University Medical Center and Radboud Institute for Health Sciences, Nijmegen, the Netherlands.

^4^Centre for Heart Rhythm Disorders, The University of Adelaide and Royal Adelaide Hospital, Adelaide, Australia.

^5^Department of Biomedical Sciences, Faculty of Health and Medical Sciences, University of Copenhagen, Copenhagen, Denmark.

^6^Brigham and Women’s Hospital and Harvard Medical School, Boston, MA, USA

^7^California Pacific Medical Center Research Institute, San Francisco, CA 94107, USA

^8^Department of Medicine and Division of Epidemiology and Community Health, University of Minnesota, Minneapolis, MN, USA

^9^Center for Care Delivery and Outcomes Research, Minneapolis VA Health Care System, Minneapolis MN, USA

*Shared first authorship

**Table S1**: Cohort characteristics of the Osteoporotic Fractures (MrOS) based on arousal-related QT and RR variance (SD_QT_, SD_RR_). Data are presented as *mean ± SD* or *n* (%).

| **Variables** | **All Subjects** | **SD_QT_< 22ms**  **(Q1-Q3)** | **SD_QT_≥ 22ms**  **(Q4)** | **p-value** | **SD_RR_<182ms**  **(Q1-Q3)** | **SD_RR_≥182s**  **(Q4)** | **p-value** |
| --- | --- | --- | --- | --- | --- | --- | --- |
| Subjects (n) | 2558 | 1899 | 659 |  | 1912 | 646 |  |
| Age (years) | 76.3±5.5 | 76.3±5.6 | 76.1±5.4 | 0.314 | 76.2±5.6 | 76.5±5.4 | 0.361 |
| White(n) | 2336(91.3) | 1738(91.5) | 598 (90.7) | 0.595 | 1750(91.5) | 586 (90.7) | 0.578 |
| African-American(n) | 79 (3.1) | 46 (2.4) | 33 (5.0) | **0.002** | 54 (2.8) | 25 (3.9) | 0.231 |
| Asian(n) | 79 (3.1) | 65 (3.4) | 14 (2.1) | 0.126 | 57 (3.0) | 22 (3.4) | 0.684 |
| Other(n) | 64 (2.5) | 50 (2.6) | 14 (2.1) | 0.564 | 51 (2.7) | 13 (2.0) | 0.438 |
| **Bodyweight** | | | | | | | |
| BMI (kg∙m^-2^) | 27.2±3.8 | 27.0±3.7 | 27.8±4.1 | **<0.001** | 27.0±3.6 | 27.6±4.2 | **0.001** |
| Overweight(n) | 1276(49.9) | 934 (49.2) | 342 (51.9) | 0.248 | 960 (50.2) | 316 (48.9) | 0.601 |
| Obese(n) | 525 (20.5) | 375 (19.7) | 150 (22.8) | 0.111 | 372 (19.5) | 153 (23.7) | **0.025** |
| **Cardiac assessment** | | | | | | | |
| Afib (%) | 258 (10.1) | 156 (8.2) | 102(15.2) | **<0.001** | 137 (7.2) | 121(18.7) | **<0.001** |
| SBP (mmHg) | 126.5±17 | 126.6±17 | 126.1±16 | 0.530 | 126.2±17 | 127.3±16 | 0.160 |
| DBP (mmHg) | 67.5±9.4 | 67.6±9.5 | 67.3±9.1 | 0.485 | 67.5±9.5 | 67.4±9.2 | 0.702 |
| **Lifestyle** | | | | | | | |
| Smoking |  |  |  |  |  |  |  |
| Never(n) | 1019 (40) | 779 (41.0) | 240 (36.4) | **0.042** | 781 (40.8) | 238 (36.8) | 0.079 |
| Past(n) | 1489(58.2) | 1087 (57.2) | 402 (61.0) | 0.101 | 1096 (57.3) | 393 (60.8) | 0.129 |
| Current(n) | 50 (1.9) | 33 (1.7) | 17 (2.6) | 0.237 | 35 (1.8) | 15 (2.3) | 0.538 |
| Current alcohol consumers(n) | 1687 (66) | 1286 (67.7) | 401 (60.8) | **0.002** | 1260 (65.9) | 427 (66.1) | 0.965 |
| PASE score | 146.4±71.2 | 148.9±71.3 | 139.3±70.6 | **0.003** | 148.0±72.1 | 140.6±68.1 | **0.016** |
| **Medical History** | | | | | | | |
| Stroke(n) | 87 (3.4) | 62 (3.3) | 25 (3.8) | 0.603 | 63 (3.3) | 24 (3.7) | 0.702 |
| CAD/MI(n) | 418 (16.3) | 285 (15.0) | 133 (20.2) | **0.002** | 306 (16.0) | 112 (17.3) | 0.465 |
| CHF(n) | 151 (5.9) | 96 (8.3) | 55 (8.3) | **0.002** | 95 (5.0) | 55 (8.5) | **0.002** |
| TIA(n) | 233 (9.1) | 175 (9.2) | 58 (8.8) | 0.810 | 168 (8.8) | 65 (10.1) | 0.371 |
| Asthma(n) | 202 (7.9) | 148 (7.8) | 54 (7.7) | 0.807 | 144 (7.5) | 58 (9.0) | 0.274 |
| COPD(n) | 136 (5.3) | 85 (4.5) | 51 (7.7) | **0.002** | 84 (4.4) | 52 (8.0) | **<0.001** |
| HTN(n) | 1271(49.7) | 936 (49.3) | 335 (50.1) | 0.523 | 951 (49.7) | 320 (49.5) | 0.965 |
| Diabetes(n) | 328 (12.8) | 228 (12) | 100 (15.2) | **0.043** | 240 (12.6) | 88 (13.6) | 0.525 |
| Parkinson(n) | 27 (1.1) | 16 (0.8) | 11 (1.7) | 0.117 | 18 (0.9) | 9 (1.4) | 0.454 |
| **Overnight Polysomnography** | | | | | | | |
| AHI (h^-1^) | 19.9±12.8 | 19.9±12.8 | 20.3±12.8 | 0.495 | 19.4±12.3 | 21.7±14.1 | **0.001** |
| AI (h^-1^) | 25.1±12.5 | 24.8±12.2 | 25.9±13.3 | 0.068 | 24.8±12.0 | 26.0±13.8 | **0.026** |
| AB (%) | 6.6±3.2 | 6.5±3.3 | 6.8±3.5 | 0.055 | 6.5±3.1 | 6.9±3.9 | **0.002** |
| PLMI (h^-1^) | 10.6±10 | 10.6±10.1 | 10.6±9.7 | 0.879 | 10.7±10.6 | 10.3±7.8 | 0.318 |
| MRR (min^-1^) | 14.8±1.9 | 14.7±1.8 | 15.1±1.7 | **<0.001** | 14.7±1.8 | 15.0±1.9 | **<0.001** |
| T90 (min) | 14.2±32.6 | 11.3±27.1 | 22.3±44.0 | **<0.001** | 11.5±28.1 | 22.0±42.3 | **<0.001** |

Afib: atrial fibrillation; BMI: body mass index; SBP: systolic blood pressure; DBP: diastolic blood pressure; PASE: physical activity scale for elderly; CAD: coronary artery disease; MI: myocardial infarction; CHF: congestive heart failure; TIA: transient ischemic attack; COPD: chronic obstructive pulmonary disease; HTN: hypertension; AHI: apnoea/hypopnoea index; AI: arousal index; AB: arousal burden; MRR: mean respiratory rate; T90:  time of sleep spent below 90% oxygen saturation.

|  | **All-cause mortality**  **(n = 865)** | | | | **Cardiovascular mortality**  **(n = 287)** | | | | **Non-cardiovascular mortality**  **(n = 578)** | | | |
| --- | --- | --- | --- | --- | --- | --- | --- | --- | --- | --- | --- | --- |
|  | **Univariate analysis** | | **Multivariable analysis** | | **Univariate analysis** | | **Multivariable analysis** | | **Univariate analysis** | | **Multivariable analysis** | |
|  | HR (95% CI) | p | HR (95% CI) | p | HR (95% CI) | p | HR (95% CI) | p | HR (95% CI) | p | HR (95% CI) | p |
| QTVi (SD) | 1.12 (1.05-1.20) | **<0.001** | 1.10 (1.03-1.19) | **0.006** | 1.30 (1.15-1.46) | **<0.001** | 1.26 (1.12-1.43) | **<0.001** | 1.05 (0.96-1.13) | 0.291 | 1.04 (0.95-1.13) | 0.409 |
| QTVi > 0.42 | 1.23 (1.06-1.42) | **0.006** | 1.16 (0.99-1.35) | 0.053 | 1.45 (1.31-1.85) | **0.003** | 1.32 (1.02-1.69) | **0.036** | 1.12 (0.94-1.35) | 0.195 | 1.09 (0.90-1.32) | 0.379 |

**Table S2:** Association of the QT time interval variability index at the onset of arousals with all-cause and cardiovascular mortality in participants with no history of atrial fibrillation. Multivariable analysis was adjusted for age, history of stroke, myocardial infarction/coronary artery disease, congestive heart failure, transient ischemic attack, diabetes, hypertension, chronic obstructive pulmonary disease, asthma, mean heart rate, mean respiratory rate, physical activity scale for elderly, systolic and diastolic blood pressure, time of sleep spent below 90% oxygen saturation, body mass index, apnea-hypopnea index, arousal index, average corrected QT, arousal burden and drink and smoking habit.

CI: confidence interval.; QTVi: QT variability index.

#
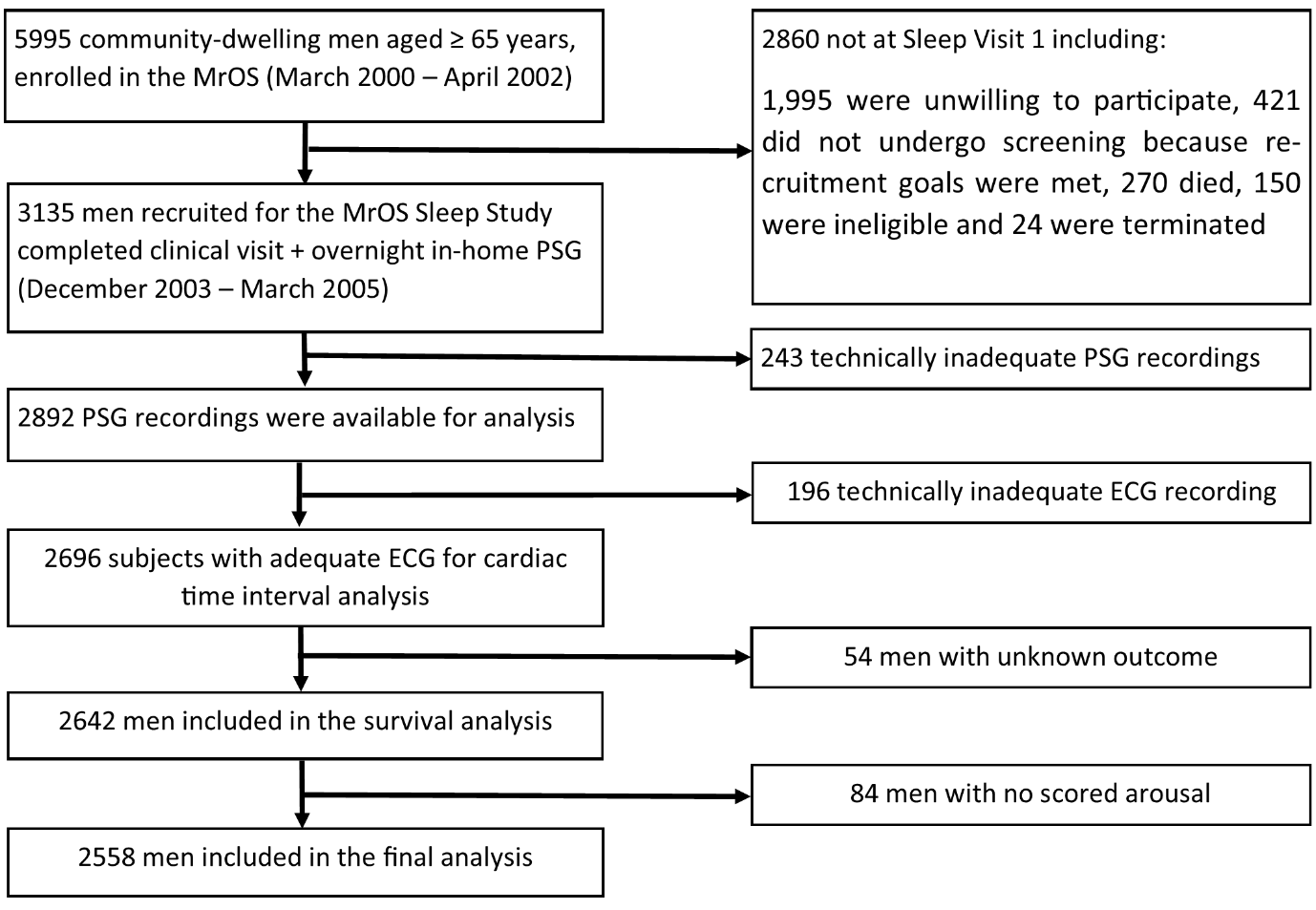


**Figure S1**: Flow charts of participants included in the analysis of arousal-related cardiac interval variability for the Osteoporotic Fractures in Men Study (MrOS).


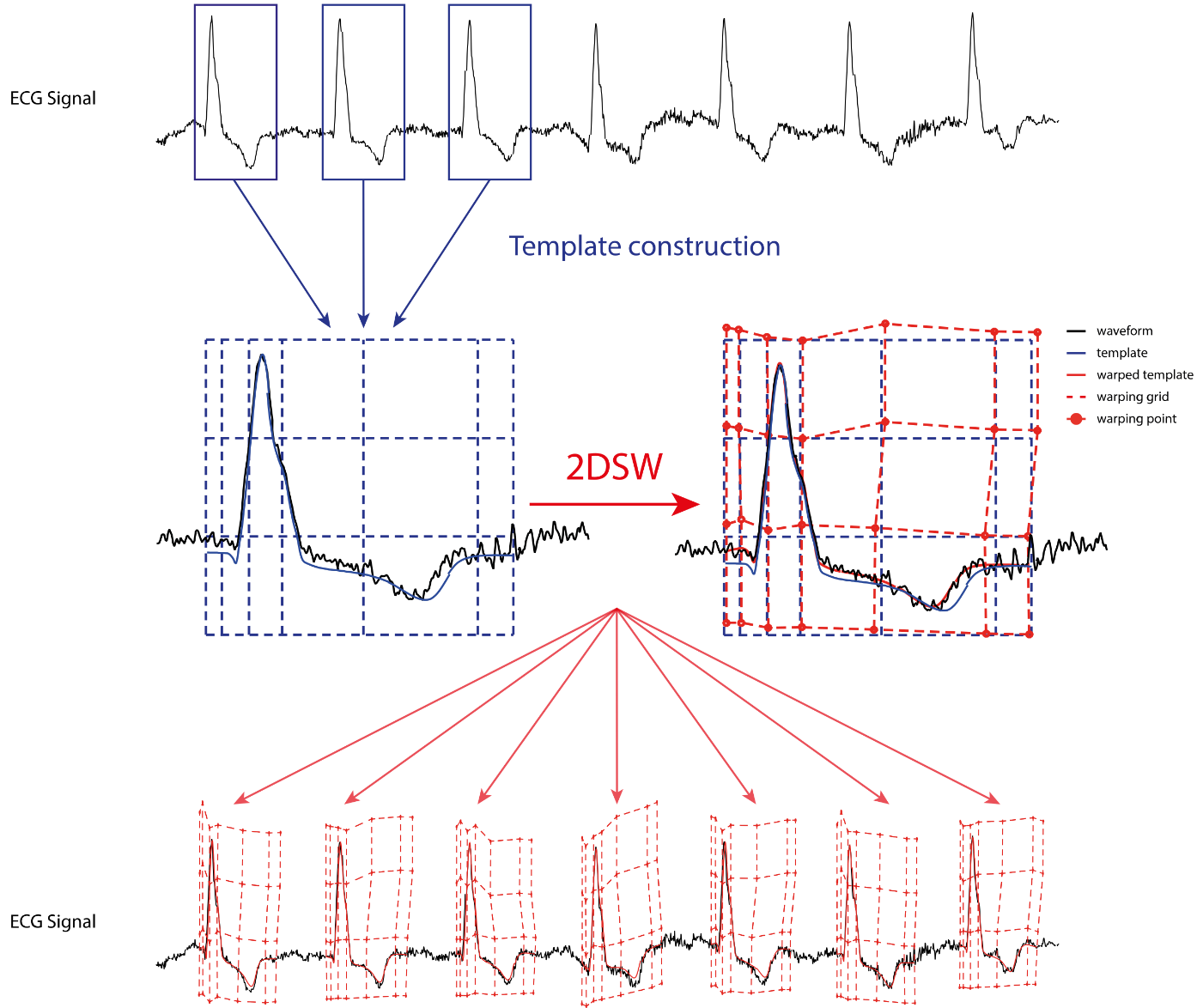


**Figure S2**: Schematic illustration of the two-dimensional signal warping (2DSW) used for measuring beat-to-beat changes in QT interval (1). The algorithm first generates a template beat based on ensemble averaging of relatively noise-free beats. The QT interval on the template is annotated in a semi-automated fashion. The template is then adapted to each waveform under consideration, exploiting 2DSW. In brief, a 2D mesh of warping points is superimposed on the template beat. These warping points are sequentially shifted in x- and y-directions, minimizing the Euclidean distance between segments of the template and the waveform.

**
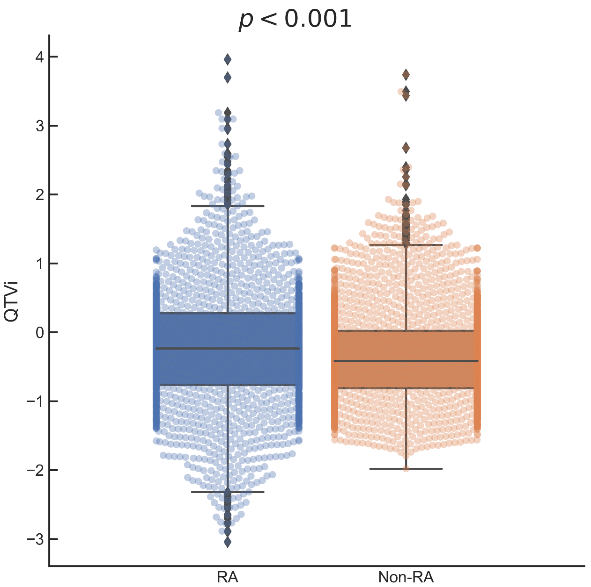
**

**Figure S3:** A comparison of the distribution of the QT variability in (QTVi) in respiratory arousals (RA) and non-respiratory arousals (Non-RA). P-value shows t-test results.


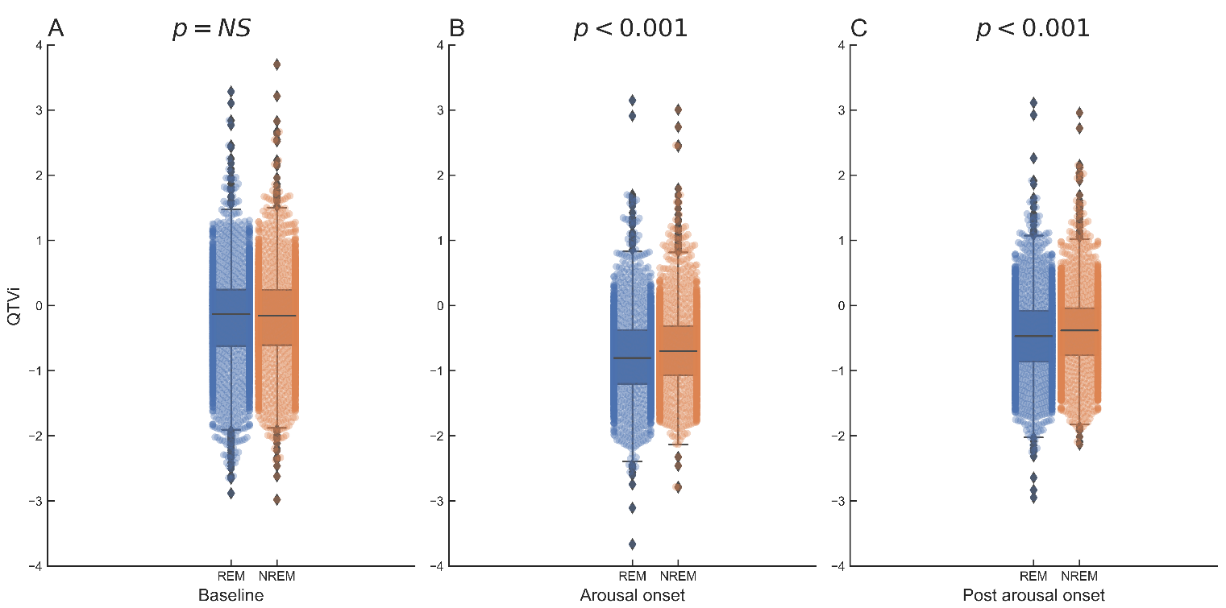


**Figure S4:** QT variability index obtained in rapid eye movement (REM) sleep and with non-rapid eye movement sleep (NREM) at baseline (A), arousal onset (B), and post arousal onset (C). P-values show t-test results.

**
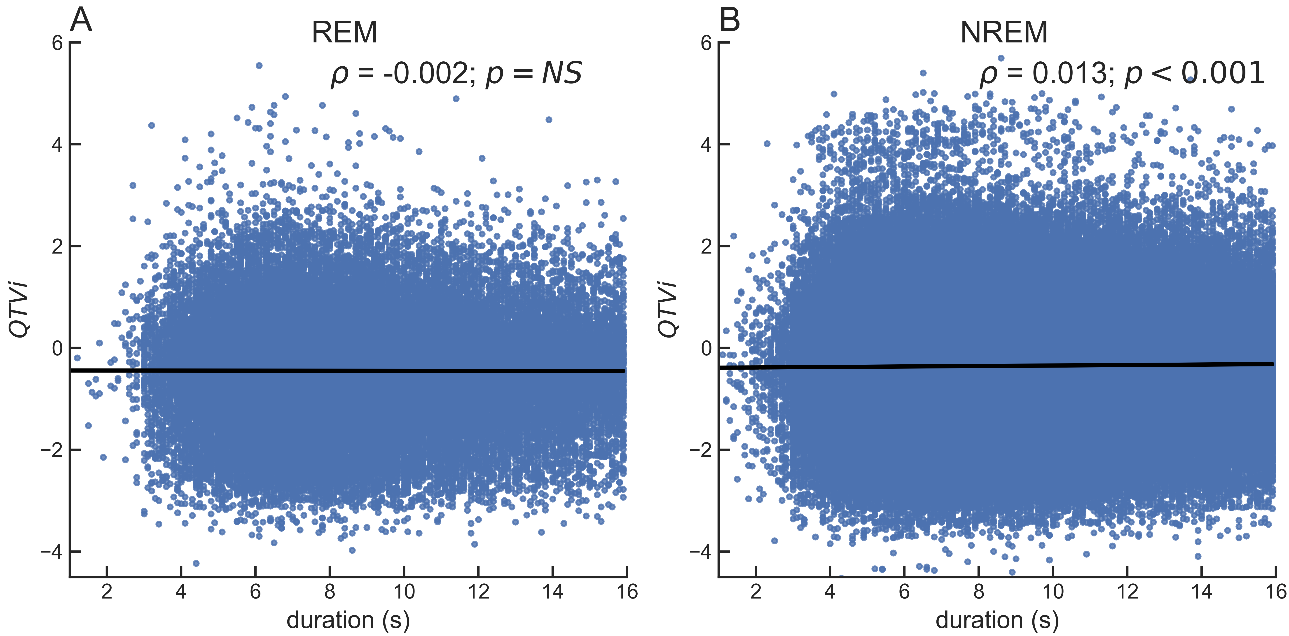
**

**Figure S5:** QT variability index during arousal onset versus arousal duration in REM sleep (A) and NREM sleep (B).


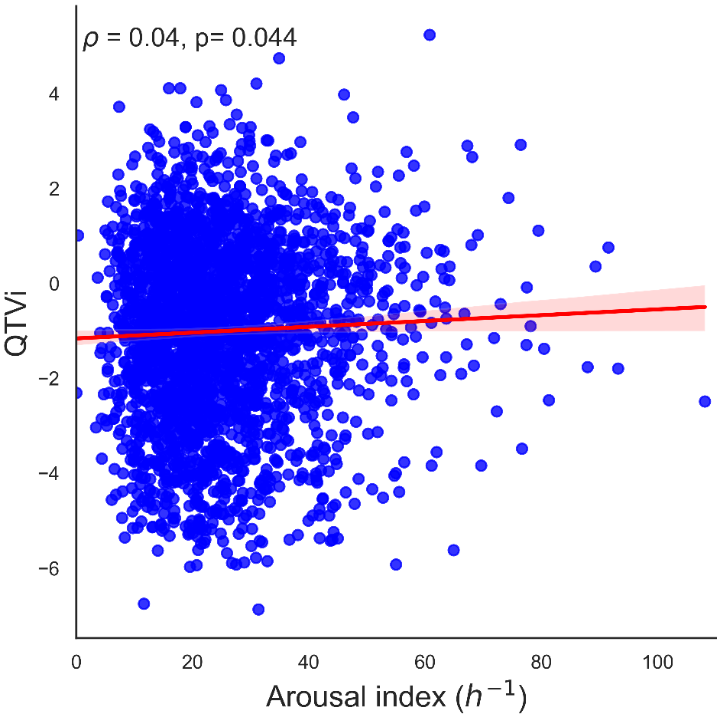


**Figure S6:** Association between QT variability index (QTVi) and arousal index.


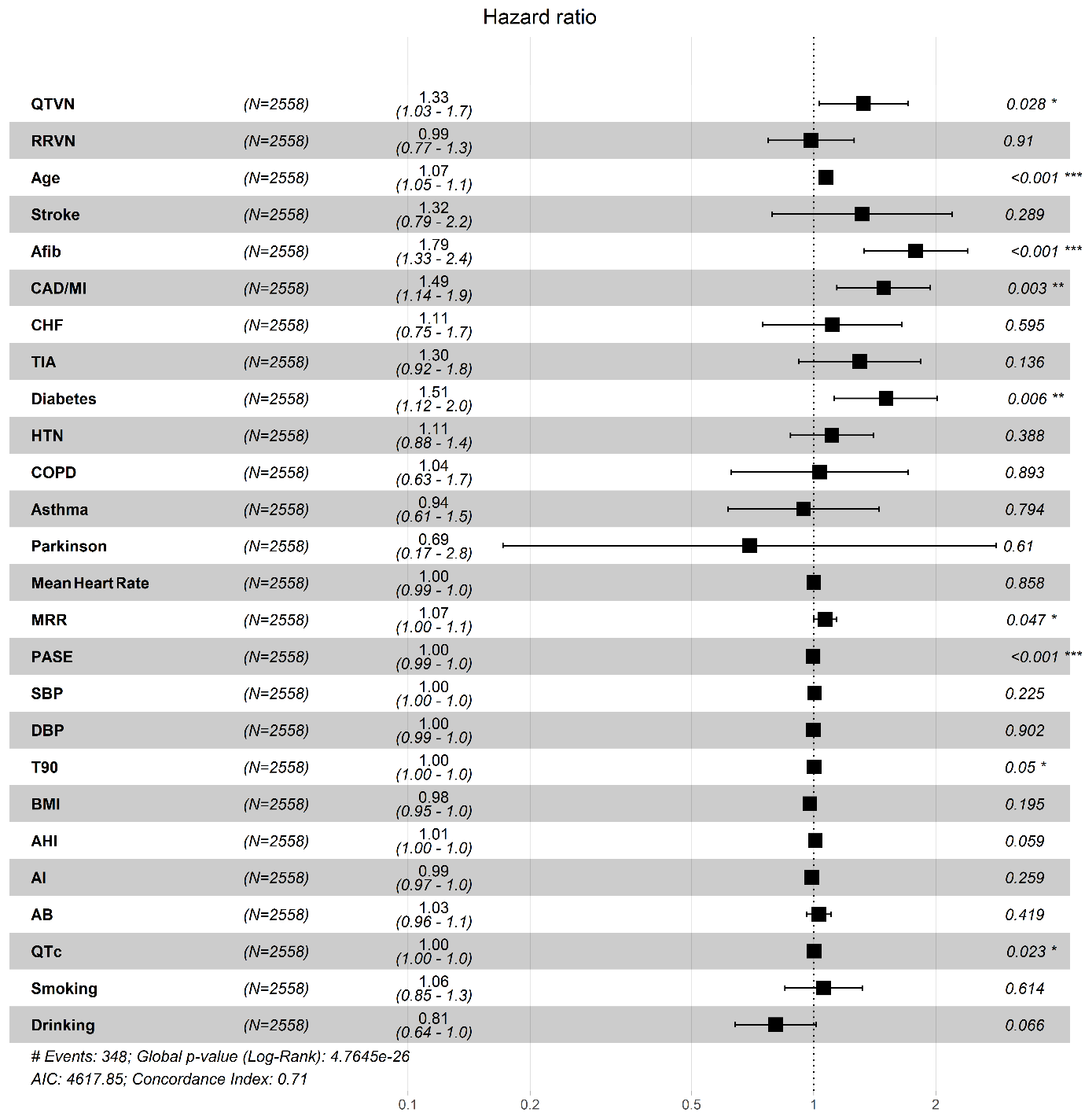


**Figure S7:** Forrest plot of covariates included in the Cox regression model for predicting CV mortality using QTVi during arousal. The QT variability component (QTVN) and RR variability component (RRVN) of QTVi were entered separately. $QTVN= \frac{{SD}_{QT}^{2}}{M_{QT}^{2}}$ and $RRVN= \frac{{SD}_{RR}^{2}}{M_{RR}^{2}}$.

**
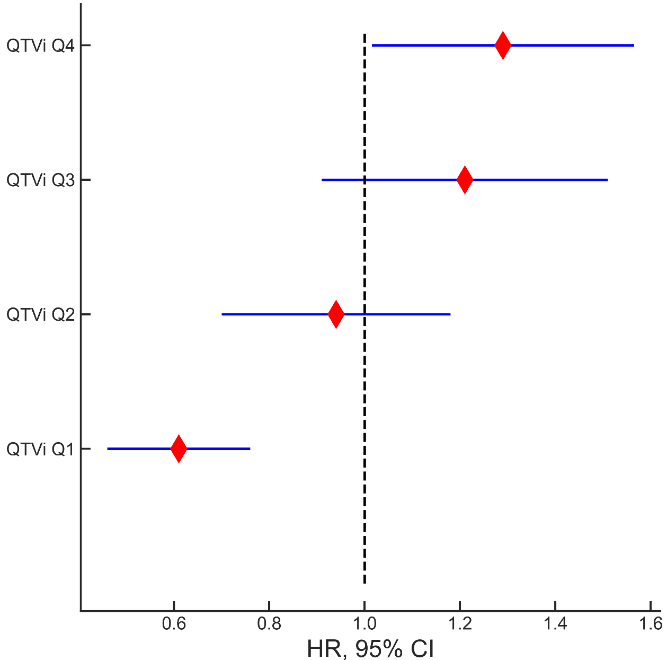
**

**Figure S8:** Hazard ratios for cardiovascular mortality for QT variability index (QTVi) quartiles at arousal onset. The Cox regression models and adjusted for age, history of stroke, myocardial infarction/coronary artery disease, atrial fibrillation, congestive heart failure, diabetes, hypertension, chronic obstructive pulmonary disease, asthma, mean heart rate, mean respiratory rate, physical activity scale for elderly, systolic and diastolic blood pressure, time of sleep spent below 90% oxygen saturation, body mass index, apnea-hypopnea index, arousal index, average corrected QT, arousal burden and drink and smoking habit.

**
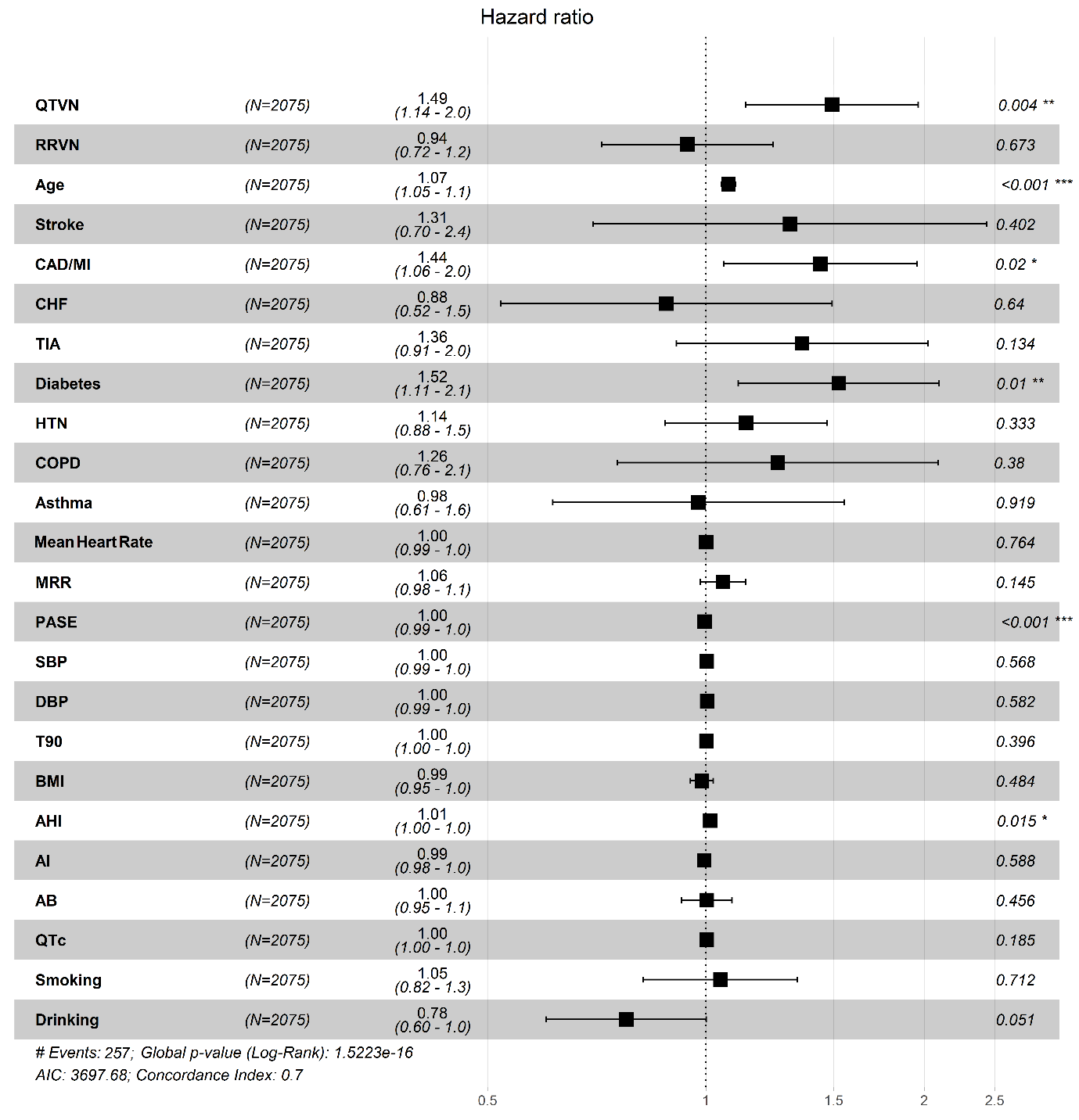
**

**Figure S9:** Forrest plot of covariates included in the Cox regression model for predicting CV mortality using QTVi during arousal and excluding participants with a history of AF. The QT variability component (QTVN) and RR variability component (RRVN) of QTVi were entered separately. $QTVN= \frac{{SD}_{QT}^{2}}{M_{QT}^{2}}$ and $RRVN= \frac{{SD}_{RR}^{2}}{M_{RR}^{2}}$.

# References

1 https://commons.wikimedia.org/w/index.php?curid=64591666
